# Genome-Wide Screening for Genes Associated with FK506 Sensitivity in Fission Yeast

**DOI:** 10.1371/journal.pone.0023422

**Published:** 2011-08-05

**Authors:** Yan Ma, Weijuan Jiang, Qingbin Liu, Sayomi Ryuko, Takayoshi Kuno

**Affiliations:** Division of Molecular Pharmacology and Pharmacogenomics, Department of Biochemistry and Molecular Biology, Kobe University Graduate School of Medicine, Kobe, Japan; Texas A&M University, United States of America

## Abstract

We have been studying calcineurin signal transduction pathway in fission yeast *Schizosaccharomyces pombe* (*S. pombe*) by developing a genetic screen for mutants that show hypersensitivity to the immunosuppressive calcineurin inhibitor FK506 (tacrolimus). In the present study, to identify nonessential genes that are functionally related to the calcineurin signaling pathway, we performed a genome-wide screen of 3004 haploid deletion strains and confirmed 72 deletion strains to be FK506 sensitive. These 72 genes are classified into nine functional groups to include membrane trafficking (16 genes), signal transduction (10 genes), ubiquitination (8 genes), chromatin remodeling (6 genes), cytokinesis (4 genes), ribosomal protein (3 genes), RNA binding protein (3 genes), and a variety of other known functions (17 genes) or still unknown functions (5 genes) in the biological system. In our previous screening of FK506-sensitive mutants we isolated several membrane-trafficking mutants showing defective cell wall integrity. Here, we further examined the vacuolar fusion, the v-SNARE synaptobrevin Syb1 localization, and the sensitivity to the β-glucan synthase inhibitor micafungin in these 72 FK506-sensitive strains. Results showed that 25 deletion strains exhibited abnormal vacuole fusion, 19 deletion strains exhibited Syb1 mislocalization, and 14 deletion strains exhibited both abnormal vacuole fusion and Syb1 mislocalization, while 42 deletion strains showed both normal vacuole fusion and Syb1 localization. Likewise, 16 deletion strains showed sensitivity to micafungin. Altogether, our present study indicates that calcineurin mediates a plethora of physiological processes in fission yeast, and that calcineurin is extensively involved in cross-talk between signaling pathways.

## Introduction

Calcineurin is a highly conserved calcium-dependent serine/threonine protein phosphatase. As a key signal transduction molecule, calcineurin mediates the Ca^2+^-dependent signaling to a wide variety of cellular responses. In mammals, calcineurin regulates a variety of physiological processes including T-cell activation, cardiac muscle development, skeletal muscle fiber-type switching, apoptosis, long-term potentiation in learning and memory, neuronal plasticity and oxidative stress [1]–[5]. Calcineurin is also involved in some pathophysiological processes such as cardiac hypertrophy, ischemia-reperfusion injury, and chronic allograft nephropathy [6]–[8].

In budding yeast *Saccharomyces cerevisiae* (*S. cerevisiae*), calcineurin is required for response to environmental stress [9], [10], endoplasmic reticulum (ER) stress [11] and cell wall damage [12]. Transcription factor Crz1 functions as a key downstream target of calcineurin [13]. In addition to transcription, calcineurin modifies the distribution of Hph1p within ER and is required for pH stress responses [14]. Slm1p and Slm2p are reported to be two novel calcineurin substrates that are required for heat stress-induced endocytosis of uracil permease [15].

In fission yeast *Schizosaccharomyces pombe* (*S. pombe*), calcineurin knockout cells showed defects in cell polarity, mating and spindle pole body positioning [16]. Calcineurin also acts antagonistically with the Pmk1 MAP1 kinase in chloride ion homeostasis [17]–[19]. In addition, we demonstrated that calcineurin activates at least two distinct signaling branches, *i.e.* the transcription factor Prz1-dependent branch that regulates the expression of the Pmc1 Ca^2+^ pump and an unknown pathway that functions antagonistically with the Pmk1 MAP kinase pathway [20].

Studies on the biological function of calcineurin have been greatly facilitated by the use of the immunosuppressive drugs FK506 (tacrolimus) and cyclosporin A, which have been widely used to prevent graft rejection after organ transplantation and have been increasingly used in the management of autoimmune diseases. FK506 is a known calcineurin inhibitor. We have been studying calcineurin signal transduction pathway in *S. pombe* by analyzing FK506-sensitive mutants. We demonstrated that calcineurin is implicated in cytokinesis, septation initiation network, membrane trafficking, and ion transport [21]–[25]. In the present study, we performed a genome-wide screen of fission yeast nonessential deletion strains to search for the genes in which gene deletion causes sensitivity to FK506, and we found that these genes encode proteins that are involved in various physiological functions such as membrane trafficking, signal transduction, ubiquitination, chromatin remodeling, cytokinesis, ribosomal protein, RNA binding, and a variety of other well-known functions or still unknown functions in the biological system. Altogether, our present study provides valuable insights on a complex signaling network in which calcineurin cross talks with other signaling pathways.

## Materials and Methods

### *S. pombe* Nonessential Gene Knockout Library

Heterozygous diploid deletion strains were constructed by Bioneer Corporation and Korea Research Institute of Biotechnology and Bioscience (http://pombe.bioneer.co.kr/). These deletion strains were generated with a genetic background of h^+^/h^+^ *ade6-M210*/*ade6-M216 leu1-32*/*leu1-32 ura4-D18*/*ura4-D18* using PCR-based deletion method [26]. The haploid deletion library used in this study consists of 3004 nonessential genes, each of which carries a defined deletion of a characterized or a putative nonessential open reading frame (ORF) replaced with the *kanMX4* cassette. Deletion of the target ORF was screened by G418 antibiotic selection.

### Media, Genetic and Molecular Biology methods

The complete medium YPD (yeast extract-peptone-dextrose) and the minimal medium EMM (Edinburgh minimal medium) have been described previously [27]. YPD plates are supplemented with 225 mg/l adenine to produce YPDA (yeast peptone dextrose adenine) plates. YE plates (0.5% yeast extract, 3% glucose, 2% agar) are supplemented with 225 mg/l adenine, histidine, leucine, uracil and lysine to produce YES (yeast extract with supplements) plates. Gene disruptions are abbreviated by the gene preceded by Δ (for example, Δ*ryh1*). Proteins are denoted by Roman letters and only the first letter is capitalized (for example, Ryh1).

### Genome-wide Screen for FK506-Sensitive Deletion Mutants

The deletion mutant library was frozen at −80°C in 96-well microtitre plates in 30% glycerol in liquid YES medium. Prior to performing the experiment, the library was transferred to YPDA plates at 27°C. The log-phase cells were streaked onto YPDA plates with or without 0.5 µg/ml FK506 (gift from Astellas Pharma Inc. Japan) and incubated at 27°C for 5 days.

### Localization of GFP-Syb1

The plasmid containing GFP-tagged Syb1, the synaptobrevin equivalent in fission yeast [28], was constructed under the thiamine-repressible *nmt1* promoter as described previously [29]. Expression was repressed by the addition of 4 µm thiamine to EMM plus adenine and uracil. The log-phase cells harboring pREP1-GFP-Syb1 were observed as described previously [29].

### Vacuole Fusion

The cells were grown to exponential phase in liquid YES medium at 27°C, harvested and washed twice with distilled water. Then the cells were resuspended in 1 ml distilled water and incubated at 27°C for 60 min. The distilled water-treated cells were harvested by centrifugation at 15000× g for 1 min at 4°C to remove excess distilled water, then placed on ice, and immediately examined under a microscope.

### Miscellaneous Methods

The differential interference contrast (DIC) and fluorescence microscopy images were recorded digitally on a Zeiss Axiophot microscope equipped with a SPOT-2 camera, and were processed with CorelDRAW software. Calcineurin-dependent response element (CDRE)-reporter activity was measured as described previously [30]. The extracellular and total acid phosphatase activities were determined as described previously [25], and the acid phosphatase secretion index was calculated as the ratio between the extracellular and the total activity and thus represents digits without units. Database searches were performed using the Sanger Center *S. pombe* data base search service (www.sanger.ac.uk).

## Results and Discussion

### Identification of genes required for growth upon FK506 exposure

To identify nonessential genes associated with increased sensitivity to FK506, we performed a genome-wide screen. First, we assessed the growth of Δ*ryh1* and Δ*ric1* cell, which showed FK506 sensitivity in our previous study [31], on YES plus FK506 or YPDA plus FK506 plates. The results showed that Δ*ryh1* and Δ*ric1* cells completely failed to grow on YPDA plus FK506 plates, whereas the cells partially grew on YES plus FK506 plates (data not shown). Therefore, we used YPDA plus FK506 plates as test plates. Next, we compared the growth of the wild-type cells with the 3004 deletion cells on YPDA and YPDA plus FK506 plates.

**Figure 1:**
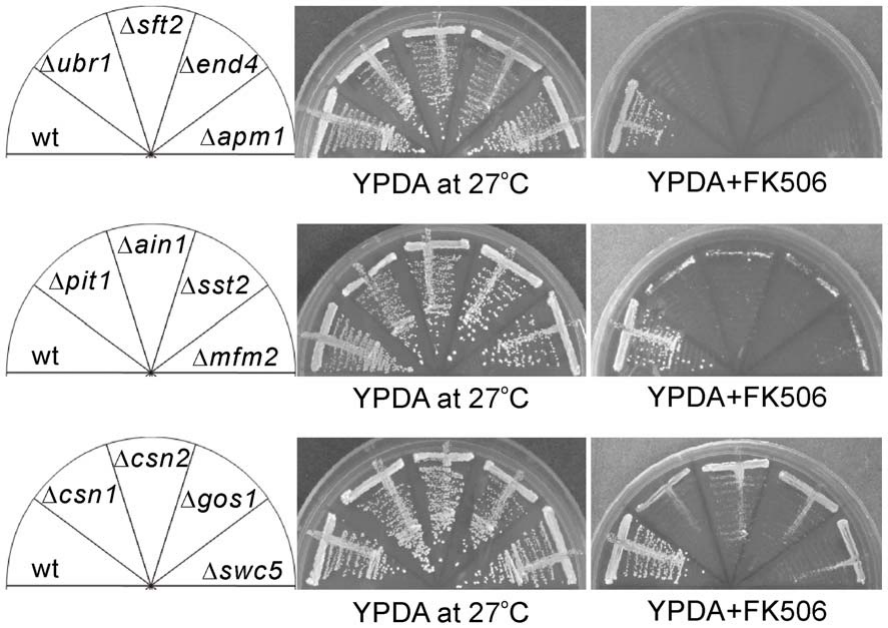
Representative growth pattern of the *S. pombe* deletion mutants in the presence of FK506. The log-phase wild-type (wt) and deletion cells as indicated were streaked onto YPDA plates with or without 0.5 µg/ml FK506 and incubated at 27°C for 5 days.

This screen resulted in the isolation of 72 deletion strains that displayed varying levels of sensitivity to FK506. The severity of growth inhibition by FK506 was scored as follows: severe (+++) indicating that the cells completely failed to growth on the FK506 containing plates (Figure 1A, upper panel), moderate (++) indicating that tiny colonies were observed to grow on the FK506-containing plates (Figure 1A, middle panel), and mild (+) indicating that colonies were observed to grow on FK506-containing plates, however, the size of the colonies were significantly smaller than that of the wild-type cell (Figure 1A, lower panel). Among the 72 FK506-sensitive mutants, 20 mutants were severely sensitive (+++), 24 were moderately sensitive (++), and 28 were mildly sensitive (+).

The 72 genes were grouped according to their functions (Table S1). The largest group consisted of genes involved in membrane trafficking (16/72 = 22.2%), the second and third largest groups consisted of genes involved in signal transduction (10/72 = 13.8%) and ubiquitination (8/72 = 11.1%), respectively. Other groups consisted of genes involved in chromatin remodeling, cytokinesis, ribosomal protein, RNA binding, and there were also a variety of genes with other known or unknown functions in the biological system. For each gene listed in Table S1, the systematic name, common name of the gene from *S. pombe* (if available), along with a brief description of the function of each gene product were also indicated. For convenience, we named the genes after their *S. cerevisiae* counterparts as the common name of the gene from *S. pombe* is not available.

### Membrane trafficking mutants that showed sensitivity to FK506

Our previous screen for FK506-sensitive mutants that require calcineurin activity for their growth resulted in the isolation of 4 membrane trafficking defective mutants, namely *its5/ypt3-i5* [32], *cis1-1/apm1-1* [29], *its6/ryh1-i6* [23], and *its11/gdi1-i11* [24]. In the present screening, 16 genes or 22.2% (16/72 genes) of the FK506-sensitive mutants showed defects that perturb intracellular membrane trafficking. Table 1 lists the intracellular localization and intracellular transport steps that these genes are involved in based on our previous reports [23], [29], [33] and on the *S. pombe* GeneDB (http://old.genedb.org/genedb/pombe/). We summarized the characterization of these 16 genes as follows. Firstly, five genes encode adaptins, specifically, two genes encode AP-1 (adaptor protein complex-1) subunits (Apm1 and Apl4), two other genes encode AP-3 subunits (Aps3 and Apl6), and one gene encodes a Golgi-localized gamma adaptin ear-containing ARF-binding (GGA) protein family adaptin (named Gga1). Secondly, six genes (Apm1, Apl4, Gga1, Ryh1, Sft2, and Gos1) localized to the dot-like structures, which mainly represent Golgi/endosomes. Two other genes (Erv15 and Erp2) localized to ER. Lastly, most of these genes are involved in the transport from the Golgi to endosomes/vacuoles, while a small proportion of these genes are involved in ER to Golgi transport (Gos1, Erv15, and Erp2), vacuole protein sorting (Vps45, Vps71, and Vps1302), and endocytosis (End4 and Myo1).

**Table 1:** Summary of the localization, vesicle-mediated transport steps, vacuole fusion, and Syb1 localization in membrane trafficking defective mutants.

Function category/Gene name	Localization	Vesicle-mediated transport steps	Vacuole fusion	Syb1 localization/Group classification*
Adaptin				
apm1	Golgi/endosomes [29]; cell tips and site of septum formation; SPBhttp://www.riken.jp/SPD/33/33B01.html	Golgi-to-endsome; endosome-to-Golgi	abnormal	abnormal(Group II)
apl4	Golgi/endosomes [33] http://www.riken.jp/SPD/38/38E09.html	Golgi-to-endsome;endosome-to-Golgi	abnormal	abnormal(Group II)
aps3	cytoplasmic dots (our unpublished data)	Golgi-to-vacuole	normal	normal(Group I)
apl6	cytoplasmic dots (our unpublished data)	Golgi-to-vacuole	normal	normal(Group I)
gga1	Golgihttp://www.riken.jp/SPD/27/27F01.html	Golgi-to-vacuole	abnormal	normal(Group I)
Small GTPase				
ryh1	cytoplasmic dots (Golgi/endosomes) [23]	endosome-to-Golgi	abnormal	abnormal(Group IV)
gyp1	cytoplasmic dotshttp://www.riken.jp/SPD/25/25A10.html	regulation of Rab GTPase	abnormal	abnormal(Group III)
Vacuole sorting protein				
vps71	nucleus>>cytosol; faint nuclear dots http://www.riken.jp/SPD/05/05G02.html	vacuolar protein sorting	normal	normal(Group I)
vps1302	no apparent signal http://www.riken.jp/SPD/34/34D07.html	endosome-to-vacuole; protein retention in Golgi apparatus	normal	normal(Group I)
vps45	cytoplasmic dotshttp://www.riken.jp/SPD/26/26C08.html	Golgi-to-endosome/vacuole; vesicle docking and fusion involved in exocytosis	abnormal	abnormal(Group IV)
Endocytosis				
end4	cytoplasmic dots, especially at cell tip and site of septum formation	endocytosis; membrane raft distribution	abnormal	abnormal(Group III)
myo1	cytoplasmic dots; periphery http://www.riken.jp/SPD/39/39C03.html	endocytosis; membrane raft distribution	normal	abnormal (Group III)
ER to Golgi				
erv15	ER and cytoplasmic dotshttp://www.riken.jp/SPD/04/04F12.html	ER-to-Golgi	normal	normal(Group I)
erp2	ER; Golgihttp://www.riken.jp/SPD/07/07C03.html	ER-to-Golgi	abnormal	normal (Group I)
Other functions				
sft2	Golgi; vacuole membranehttp://www.riken.jp/SPD/10/10D12.html	Golgi-to-endsome	abnormal	abnormal(Group IV)
gos1	Golgihttp://www.riken.jp/SPD/05/05F02.html	ER-to-Golgi and intra-Golgi transport	abnormal	abnormal(Group IV)

*Group I indicates that similar to wild-type cells GFP-Syb1 was visibly seen on the plasma membrane with more prominence at the growing ends of the cells.

Group II indicates that GFP-Syb1 accumulated as large dot-like structures in the cytoplasm.

Group III indicates that GFP-Syb1 localized almost evenly all around the cell surface, and the punctate structures in the cytoplasm were barely observed.

Group IV indicates that GFP-Syb1 failed to localize to the membrane and the fluorescence was weak and hazy.

**Figure 2:**
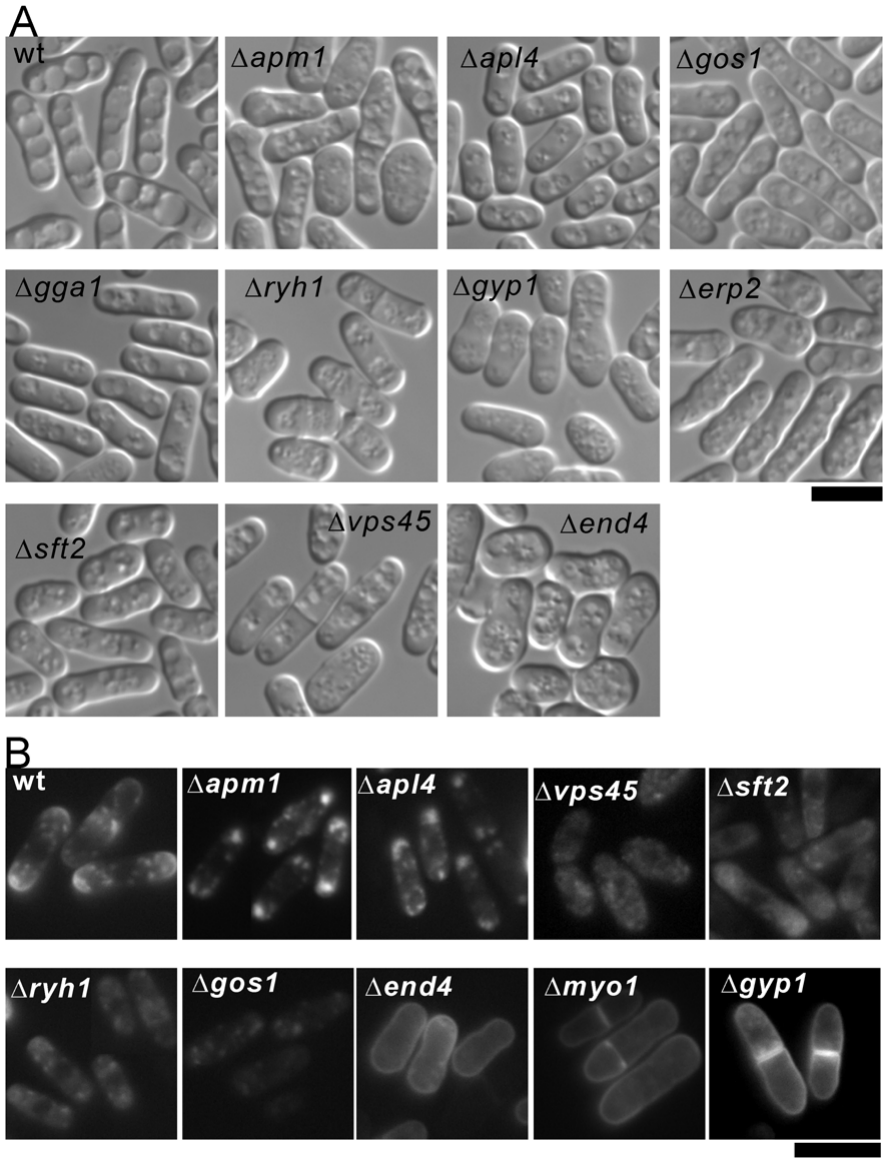
The vacuole fusion and Syb1 localization in membrane trafficking defective mutants. (A) The vacuole fusion of membrane trafficking defective mutants. The wild-type (wt) cells and membrane trafficking defective mutants were grown to early log-phase in YES media at 27°C, harvested and washed twice with distilled water. Then the cells were resuspended in 1 ml distilled water and incubated at 27°C for 60 min. The distilled water-treated cells were harvested by centrifugation at 15000× g for 1 min at 4°C to remove excess distilled water, then placed on ice, and immediately examined under a fluorescence microscope. Bar, 10 µm. (B) The Syb1 localization of membrane trafficking defective mutants. The wild-type (wt) cells and membrane trafficking defects mutants were transformed with pKB4160 (pREP1-GFP-Syb1) respectively, and the cells were spread onto EMM plus 225 mg/l adenine, 225 mg/l uracil and 4 µM thiamine. Three single colonies were individually picked up, further subcultured and grown to early log-phase on the EMM plates as described above. GFP-Syb1 was examined under the fluorescence microscope. Bar, 10 µm.

To further examine the role of these 16 genes in membrane trafficking, we investigated the vacuolar morphology and the intracellular localization of v-SNARE synaptobrevin Syb1, a Snc1p homolog [23]. On vacuolar morphology, as shown in Figure 2A, the wild-type cells had evidently large vacuoles that resulted from vacuole fusion, whereas 10 of the 16 deletion strains showed significant defects in vacuole fusion. Only 6 of the 16 genes (Aps3, Apl6, Vps1302, Vps71, Erv15 and Myo1) showed normal vacuole fusion. A summary of the characteristic features are listed in Table 1.

In group I deletion strains, similar to wild-type cells, GFP-Syb1 was visibly seen on the plasma membrane with more prominence at the growing ends of the cells (data not shown). Of the seven strains categorized in group I, two strains (Δ*erv15* and Δ*erp2*) showed defects in the transport from ER to the Golgi transport, three strains (Δ*aps3*, Δ*apl6*, and Δ*gga1*) exhibited defects in the transport from the Golgi to vacuole, and the other two strains (Δ*vps71* and Δ*vps1302*) showed defects in vacuole protein sorting (Table 1). In group II deletion strains, GFP-Syb1 accumulated as large dot-like structures in the cytoplasm as exemplified by Δ*apm1* and Δ*apl4* (Figure 2B). These two strains exhibited defects in the anterograde transport from the Golgi to the endosome or from the endosome to the plasma membrane, or they showed defects in the retrograde transport from endosome to the Golgi. In group III deletion strains, GFP-Syb1 localized almost evenly all around the cell surface, and the punctate structures in the cytoplasm were barely observed (Figure 2B, Δ*end4*, Δ*myo1*, and Δ*gyp1*). The Δ*end4* and Δ*myo1* strains were characterized to have defects in endocytosis [33]–[36]. Interestingly, knockout of the *gyp1* ^+^ gene, which encodes Rab/Ypt GTPase activating protein (GAP), also showed the accumulation of Syb1 on the plasma membrane (Figure 2B). We speculate that the Δ*gyp1* mutants have defects in endocytosis, so that Syb1 failed to be endocytosed, thus accumulating on the plasma membrane. In agreement with our hypothesis, in budding yeast it is reported that *in vitro* Gyp1p acts on Ypt51p, that encodes a Rab/Ypt GTPase required for endocytosis [37], [38]. Likewise, in fission yeast, Gyp1 may play an important role in endocytosis via the GAP activity. In group IV deletion strains, GFP-Syb1 failed to localize to the membrane and the fluorescence was weak and hazy (Figure 2B). Of the four strains categorized in group IV, two strains (Δ*vps45* and Δ*sft2*) showed defects in the transport from the Golgi to endosomes, one strain (Δ*ryh1*) exhibited defects in the recycling from the endosome to Golgi, and still another strain (Δ*gos1*) showed defects in both ER to the Golgi and intra-Golgi transport (Table 1).

To further investigate the genetic/functional interaction between these membrane trafficking mutants and calcineurin, we examined the effect of C-terminal-deleted Ppb1 (Ppb1ΔC), the constitutively active form of Ppb1, on vacuole fusion and on Syb1 localization. The results showed that Ppb1ΔC overexpression failed to suppress the defective vacuole fusion of the membrane trafficking mutants listed in Table 1 (data not shown). Also, Ppb1ΔC overexpression failed to suppress defective Syb1 localization in the Δ*apl4*, Δ*end4*, and Δ*gos1* cells (data not shown). Altogether, our results suggest that the increased calcineurin activity failed to compensate the defects in membrane trafficking, and that calcineurin and these membrane trafficking genes function in a parallel pathway to control cell wall integrity.

### Ubiquitination mutants that showed sensitivity to FK506

**Figure 3:**
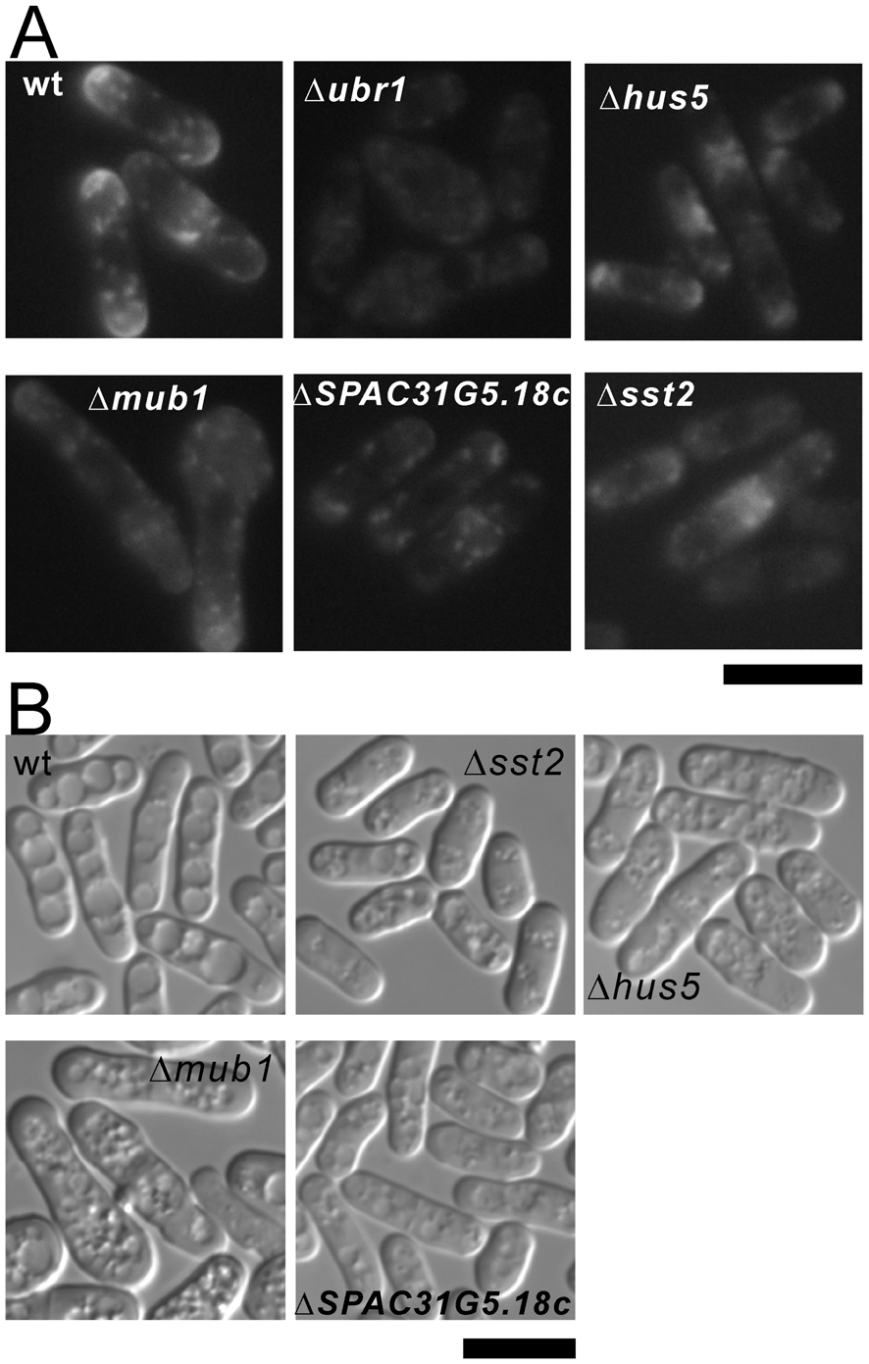
The vacuole fusion and Syb1 localization in ubiquitination defective mutants. (A) The vacuole fusion of ubiquitination defective mutants. The experiments were performed as described in Figure 2A. Bar, 10 µm. (B) The Syb1 localization of ubiquitination defective mutants. The experiments were performed as described in Figure 2B. Bar, 10 µm.

Ubiquitination is an extremely versatile control mechanism that regulates almost all aspects of cell life, including cell cycle, DNA transcription and repair, differentiation and development, apoptosis, signaling transduction and membrane trafficking. Ubiquitination plays important roles not only in the internalization of plasma membrane proteins, but also in their sorting to the different cellular destinations, namely the trans-Golgi network (TGN), endosome, lysosomes/vacuoles or for degradation by the proteosome [39]. Majority of ubiquitin-tagged molecules usually are degraded by the 26S proteasome, and a minority of the ubiquitin-tagged membrane proteins are endocytosed and degraded in the vacuole. To investigate whether the FK506 sensitivity of these ubiquitination-related gene deletion strains is mediated by membrane trafficking, we observed the Syb1 localization and the vacuole fusion. As shown in Figure 3A, in Δ*ubr1*, Δ*sst2* (SPAC19B12.10), Δ*mub1* (SPBC31F10.10c), Δ*hus5/ubc9* (SPAC30D11.13), and ΔSPAC31G5.18c cells, GFP-Syb1 failed to localize to the plasma membrane, instead GFP-Syb1 distributed throughout the intracellular space. Syb1 showed normal localization in the mutant cells of SPAC31G5.18c (encoding ubiquitin-protein ligase E3) and in the mutant cells of *csn1^+^* and *csn2^+^* (encoding COP9/signalosome complex subunit) (data not shown). In Δ*sst2*, Δ*mub1*, and Δ*hus5/ubc9* cells, the vacuoles remained numerous and small, suggesting a defect in vacuole fusion. Table 2 showed a summary of the vacuole fusion and Syb1 localization in ubiquitination defective mutants. Taken together, we speculate that the ubiquitination-related gene deletion strains displayed FK506 sensitivity mostly via a defect in membrane trafficking (Figure 3B).

**Table 2:** Summary of the vacuole fusion and Syb1 localization in ubiquitination defective mutants.

Gene name	Products	Vacuole fusion	Syb1 localization
ubr1	N-end-recognizing protein Ubr1 E3	abnormal	normal
sst2	human amsh protein homolog	abnormal	abnormal
mub1	zf-MYND type zinc finger protein	abnormal	abnormal
hus5/ubc9	SUMO conjugating enzyme	abnormal	abnormal
SPAC6B12.07c	ubiquitin-protein ligase E3	normal	normal
SPAC31G5.18c	ubiquitin family, human C1ORF55 related	abnormal	normal
csn1	COP9/signalosome complex subunit Csn1	normal	normal
csn2	COP9/signalosome complex subunit Csn2	normal	normal

### Membrane trafficking and ubiquitination mutants showed defective acid phosphatase secretion

**Figure 4:**
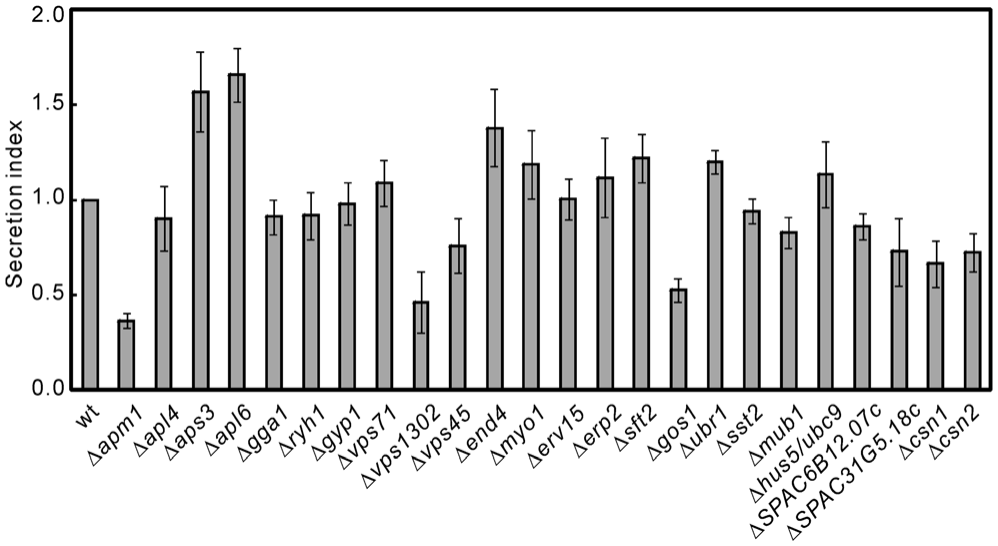
Acid phosphatase secretion in membrane trafficking and ubiquitination defective mutants. The cells as indicated were grown to exponential phase at 27°C in liquid EMM supplemented with 225 mg/l for each of the folloeing amino acid: adenine, histidine, leucine and uracil. The cells were harvested and washed twice with EMM, and then resuspended in liquid supplemented EMM with OD660 = 0.3. The extracellular and total acid phosphatase activities were determined as described in Materials and Methods, and **t**he acid phosphatase secretion index was calculated as the ratio between the extracellular and the total activity and thus represents digits without units. The error bars were calculated as the standard error from at least three independent experiments.

Our previous results showed that Δ*ryh1* and Δ*apm1* mutants showed defective acid phosphatase secretion [23], [29]. This prompted us to investigate whether acid phosphatase secretion is defective in these membrane trafficking and ubiquitination mutants. The results showed that in Δ*apm1*, Δ*vps1302* and Δ*gos1* cells the acid phosphatase secretion index was dramatically decreased to approximately 50% of that in wild-type cells (Figure 4). In Δ*csn1* and Δ*csn2* cells, the acid phosphatase secretion was moderately decreased to approximately 75% of that in wild-type cells (Figure 4). Notably, the deletion of the two subunits of the AP-3 complex, in particular Δ*aps3* and Δ*apl6*, resulted in an approximately two-fold increase in the acid phosphatase secretion index (Figure 4).

### Cell wall integrity mutants that showed sensitivity to FK506

In *S. cerevisiae* and *S. pombe*, calcineurin is required for response to cell wall damage [12], [40]. To investigate whether the 72 FK506-sensitive mutants show defects in cell wall integrity, we streaked these cells onto YPDA plate containing micafungin, an inhibitor of (1, 3)-β-D-glucan synthase. Fifteen deletion strains were confirmed to display varying levels of hypersensitivity to micafungin (Table 3). The severity of growth inhibition on YPDA plate plus 1.5 µg/ml micafungin was scored as follows: severe (+++) indicating that the cells completely failed to grow on YPDA plus 1.5 µg/ml micafungin, moderate (++) indicating that tiny colonies were observed on the micafungin-containing plates, or mild (+) indicating that colonies were observed on the micafungin-containing plates, however, the size of the colonies were significantly smaller than that of the wild-type cells (Table 3). Three mutants (Δ*aps3*, Δ*sst2*, and Δ*cyb5*) were strongly sensitive (+++), seven mutants (Δ*gyp1*, Δ*lsg1*, Δ*ryh1*, Δ*apm1*, Δ*vps45*, Δ*pal1*, and Δ*vps1302*) were moderately sensitive (++), and six mutants (Δ*apl6*, Δ*ste20*, Δ*ubr1*, Δ*rga7*, Δ*mug132*, and Δ*uge1*) were mildly sensitive (+). Previously, we speculated that the FK506 sensitivity of the membrane trafficking defective mutant may result as a consequence of the impaired cell wall integrity. However, among the 15 FK506-sensitive mutants, 8 deletion strains did not show micafungin sensitivity. Therefore, we conclude that in addition to cell wall integrity, other mechanism is involved in FK506 sensitivity. In budding yeast, it is reported that *FKS1* and *FKS2* encode the catalytic subunits of (1, 3)-β-D-glucan synthase, and the expression of *FKS2* is induced by the addition of Ca^2+^ to the growth medium in a calcineurin-dependent manner [41], [42]. This prompted us to observe whether the overexpression of the constitutively active form of Ppb1 (Ppb1ΔC) could complement the micafungin sensitivitity. The results showed that Ppb1ΔC overexpression failed to suppress the micafungin sensitivity, suggesting that the increased calcineurin activity failed to compensate the cell damage caused by micafungin.

**Table 3:** Summary of the micafungin sensitivity of FK506-sensitive mutants.

Gene name	YPDA+1.5 µg/ml micafungin
aps3, sst2, cyb5	+++
gyp1, lsg1, ryh1, apm1, vps45, pal1, vps1302	++
apl6, ste20, ubr1, rga7, mug132	+

+++ Indicates that the cells completely failed to grow on YPDA plus micafungin plates.

++ Indicates that tiny colonies were observed to grow on YPDA plus micafungin plates.

+ Indicates that colonies were observed on YPDA plus micafungin plates, however, the size of the colonies were significantly smaller than that of the wild-type cells.

### Signaling transduction mutants that showed sensitivity to FK506

As shown in Table S1, ten signaling transduction mutants were identified to display FK506 sensitivity. Three genes (Ckb1, Pit1, and Wis1) encode protein kinases (PKs), another three genes (Par1, Clp1, and Pmp1) encode protein phosphatases (PPs), and another gene Rad24 encodes a 14-3-3 family protein, which interacts with more than 200 phosphorylated proteins in mammals. Protein phosphorylation plays a crucial role in biological functions and controls nearly all cellular processes. These processes depend on the highly regulated and opposing actions of PKs and PPs, through changes in the phosphorylation of key proteins. We speculate that some PKs or PPs have common substrates with calcineurin, and that this substrate may be involved in an essential function.

Recently, it is reported that the Ste20-dependent phosphorylation modulates the dynamic localization and endocytic function of Myo1 in fission yeast [35]. This prompted us to investigate whether the localization of Syb1 in Δ*ste20* mutants is similar to that in Δ*myo1* mutants. Contrary to our expectations, in Δ*ste20* mutants GFP-Syb1 did not accumulate on the cell surface, and instead was distributed throughout the intracellular space (data not shown). We also investigated the localization of GFP-Syb1 in the other three deletion strains (Δ*sin1*, Δ*pop3* and Δ*bit61*) of the TORC2 complex components, and results showed that Δ*ste20* mutants, but not the other three TORC2 complex components, displayed Syb1 mislocalization (data not shown). Taken together, our study suggests that Ste20 plays a role in membrane trafficking in addition to its role as a member of the TORC2 complex.

### Signaling transduction mutants, ubiquitination mutants and unknown function mutants showed abnormal CDRE-reporter activity

We previously established an *in vivo* real-time monitoring system of calcineurin activity utilizing the reporter harboring the calcineurin-dependent response element (CDRE)-fused to luciferase, and showed that high extracellular CaCl_2_ and NaCl concentration caused an increase in the CDRE-reporter activity in fission yeast [30]. To investigate whether the calcineurin activity was affected in these FK506-sensitive mutants, we monitored the CDRE reporter activity in the signaling transduction mutants, ubiquitination mutants and other mutants with unknown function.

**Figure 5:**
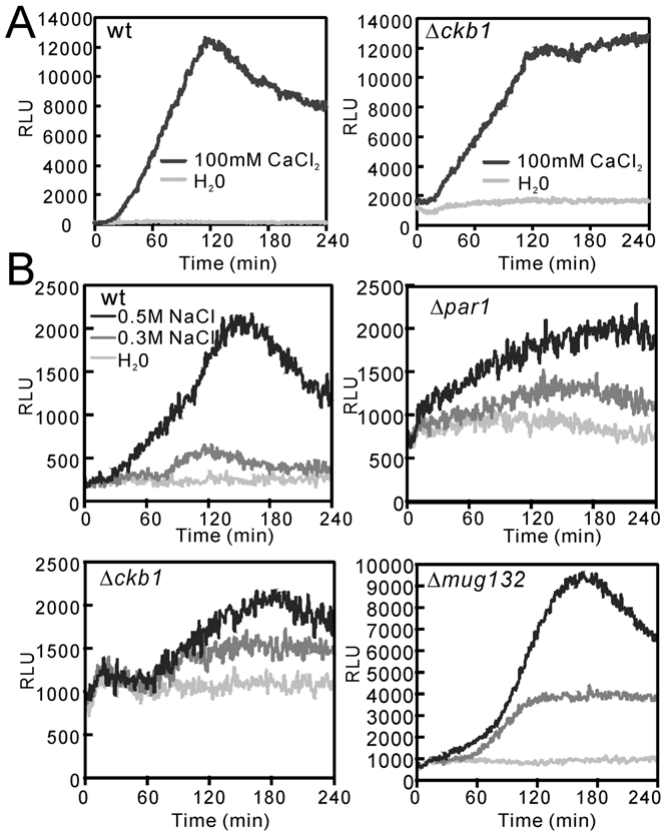
CDRE-reporter activity in FK506-sensitive mutants. The wild-type and deletion cells harboring the plasmid containing the 3xCDRE::luc(R2.2) reporter gene were grown to exponential phase in liquid EMM at 27°C. The reporter activity was monitored as described in Materials and Methods. The data shown are representative of multiple experiments.

The result showed that all of these mutants except for Δ*ckb1* showed responses that cannot be distinguished from wild-type cells to elevated extracellular CaCl_2_. In wild-type cells, elevated extracellular CaCl_2_ caused an increase in the calcineurin activity as exhibited by a peak rise and then a slow decay (Figure 5A, wt). However, in Δ*ckb1* mutants, elevated extracellular CaCl_2_ caused an increase in the calcineurin activity that exhibited a peak rise and then approached a constant high level (Figure 5A, Δ*ckb1*). Notably, the basal calcineurin activity in Δ*ckb1* cells was markedly higher than that in wild-type cells (Figure 5A), suggesting that Ckb1 is involved in the regulation of Ca^2+^/calcineurin/Prz1 pathway.

The deletion strains were classified into three groups (Table 4) according to their response to elevated extracellular NaCl. Gene deletion strains belonging to Group A showed responses that cannot be distinguished from wild-type cells (Table 4 and Figure 5B, wt). Gene deletion strains belonging to Group B showed high basal RLU with normal response to NaCl (Table 4 and Figure 5B, Δ*mug132*). Gene deletion strains belonging to Group C showed high basal RLU with low response to NaCl (Table 4 and Figure 5B, Δ*par1* and Δ*ckb1*). Of the seven mutants that showed high basal transcription activity (Table 4), 4 mutants (Δ*sst2*, Δ*ste20*, Δ*ubr1* and Δ*mug132*) also showed micafungin sensitivity, suggesting that cell wall integrity defect may result in a high transcriptional activity to compensate for the defects. Our results also suggest that Ckb1 and protein phosphatase regulatory subunit Par1 may crosstalk with Ca^2+^/calcineurin/Prz1 pathway.

**Table 4:** The 3xCDRE::luc(R2.2) reporter activity in various deletion cells upon NaCl stimulation.

Function category	Group A(normal basal RLU with normal response)	Group B(high basal RLU with normal response)	Group C(high basal RLU with low response)
Signal transduction	Δ*far8*, Δ*pmp1*, Δ*rga7*, Δ*rad24*, Δ*pit1*, Δ*wis1*, Δ*clp1*	Δ*ste20*	Δ*ckb1*, Δ*par1*
Unknown functions	Δ*ssp120*, Δ*mug113*, Δ*mde1*, Δ*SPCC592.04c*	Δ*mug132*	
Ubiquitination	Δ*mub1*, Δ*SPAC6B12.07c*, Δ*hus5/ubc9*	Δ*sde2*, Δ*ubr1*	Δ*sst2*

The wild-type and deletion cells respectively were transformed with the plasmid containing the 3xCDRE::luc(R2.2) reporter gene, and the reporter activity was monitored as described in Materials and Methods.

### Perspective on the side effects of FK506

FK506 has well-documented side effects such as infection, cardiac damage, hypertension, nephrotoxicity and various neuropsychiatric problems [43]–[45]. FK506 also increases the risk of cancer [46], [47]. However, the molecular basis for these adverse effects is not yet fully understood. In model organisms such as fission yeast, synthetic lethality has been extensively used to characterize the interactions between genes that are likely to be involved in similar processes and to uncover new molecular interactions. In cases when both mutations are null, the interpretation is that the two genes are required in parallel pathways toward a common function, and the loss of gene function is lethal. In the present study, we systematically screened FK506-sensitive mutants to identify the possible molecular component or signaling pathway underlying the therapeutic action and the unwanted effects of FK506, and we identified 72 mutations that display synthetic lethal interactions with calcineurin. It is particularly interesting to note that most of the FK506-sensitive deletion strains identified in this study were defective in various steps in membrane trafficking. Thus, normal membrane trafficking is required for FK506 resistance, and membrane trafficking mutants require calcineurin activity for their growth.

## Supporting Information

Table S1 Summary of the gene name and products of FK506-sensitive mutants.
(DOCX)
