# Supplementary material for: Genome-Wide Screening for Genes Associated with FK506 Sensitivity in Fission Yeast
Source: PLoS One. 2011 Aug 5;6(8):e23422. doi: 10.1371/journal.pone.0023422 (PMC3151288; doi:10.1371/journal.pone.0023422)
Supplement: Table S1 — Summary of the gene name and products of FK506-sensitive mutants. (DOCX) [file pone.0023422.s001.docx]

**Table S1 Summary of the gene name and products of FK506-sensitive mutants**

| **Function category/**  **FK506 sensitivity** | **Systematic Name** | **Gene Name** | **Product** | |
| --- | --- | --- | --- | --- |
| **Membrane trafficking** | | | | |
| **+++** | **SPAC1527.02** | **sft2** | **Golgi transport protein Sft2** | |
| **+++** | **SPBP16F5.07** | **apm1** | **AP-1 adaptor complex subunit Apm1** | |
| **+++** | **SPAC4C5.02c*** | **ryh1*** | **GTPase Ryh1** | |
| **+++** | **SPBC530.01** | **gyp1** | **GTPase activating protein Gyp1** | |
| **+++** | **SPAC688.11** | **end4** | **Huntingtin-interacting protein homolog** | |
| **+++** | **SPBC146.13c*** | **myo1*** | **myosin type I** | |
| **+++** | **SPAC2G11.03c** | **vps45** | **vacuolar sorting protein Vps 45** | |
| **+++** | **SPBC16C6.02c*** | **vps1302*** | **chorein homolog** | |
| **++** | **SPBC25H2.16c** | **gga1^①^** | **adaptin** | |
| **++** | **SPAC30D11.05** | **aps3** | **AP-3 adaptor complex subunit Aps3** | |
| **++** | **SPCP1E11.06** | **apl4** | **AP-1 adaptor complex gamma subunit Apl4** | |
| **++** | **SPAC2C4.05** | **erv15^①^** | **cornichon family protein** | |
| **++** | **SPAC17A5.08** | **erp2^①^** | **COPII-coated vesicle component Erp2/3/4** | |
| **+** | **SPAC23H3.06*** | **apl6*** | **AP-3 adaptor complex subunit Apl6** | |
| **+** | **SPAC4G8.10** | **gos1** | **SNARE Gos1** | |
| **+** | **SPBC29A3.05** | **vps71** | **chromatin remodeling complex subunit** | |
| **Signal transduction** | | | | |
| **+++** | **SPBC1773.01** | **far8^①^** | **striatin homolog** | |
| **+++** | **SPCC188.02** | **par1** | **protein phosphatase regulatory subunit Par1** | |
| **+++** | **SPAC1851.03*** | **ckb1*** | **CK2 family regulatory subunit** | |
| **+++** | **SPBC23G7.08c** | **rga7** | **GTPase activating protein Rga7** | |
| **+++** | **SPBC12C2.02c** | **ste20** | **Rictor homolog, Ste20** | |
| **++** | **SPAC8E11.02c** | **rad24** | **14-3-3 protein Rad24** | |
| **++** | **SPAC3C7.06c** | **pit1** | **serine/threonine protein kinase Pit1** | |
| **++** | **SPBC409.07c** | **wis1** | **MAP kinase kinase Wis1** | |
| **+** | **SPAC1782.09c** | **clp1** | **Cdc14-related protein phosphatase Clp1/Flp1** | |
| **+** | **SPBC1685.01** | **pmp1** | **dual-specificity MAP kinase phosphatase** | |
| Ubiquitination | | | | |
| **+++** | **SPBC19C7.02** | **ubr1** | **N-end-recognizing protein Ubr1** | |
| **++** | **SPAC19B12.10** | **sst2** | **human amsh protein homolog** | |
| **++** | **SPBC31F10.10c*** | **mub1^①^*** | **zf-MYND type zinc finger protein** | |
| **++** | **SPAC30D11.13** | **hus5/ubc9** | **SUMO conjugating enzyme** | |
| **+** | **SPAC6B12.07c** | **N/A** | **ubiquitin-protein ligase E3** | |
| **+** | **SPAC31G5.18c** | **sde2** | **ubiquitin family, human C1ORF55 related/**  **silencing defective protein Sde2** | |
| **+** | **SPBC215.03c** | **csn1** | **COP9/signalosome complex subunit Csn1** | |
| **+** | **SPAPB17E12.04c** | **csn2** | **COP9/signalosome complex subunit Csn2** | |
| **Chromatin remodeling** | | | | |
| **++** | **SPBC11B10.10c** | **pht1** | | **histone H2A variant H2A.Z** |
| **++** | **SPBC21C3.02c** | **dep1** | | **Sds3-like family protein Dep1** |
| **+** | **SPAC22E12.11c** | **set3** | | **histone lysine methyltransferase Set3** |
| **+** | **SPBC800.03** | **clr3** | | **histone deacetylase (class II) Clr3** |
| **+** | **SPCC576.13** | **swc5** | | **chromatin remodeling complex subunit Swc5** |
| **+** | **SPBC14C8.17c** | **spt8** | | **SAGA complex subunit Spt8** |
| **Cytokinesis** | | | | |
| **+++** | **SPBC83.18c** | **fic1** | | **C2 domain protein** |
| **++** | **SPCP1E11.04c*** | **pal1*** | | **membrane associated protein Pal1** |
| **++** | **SPAC15A10.08** | **ain1** | | **alpha-actinin** |
| **+** | **SPAC16E8.01** | **shd1/sla1** | | **cytoskeletal protein binding protein Sla1 family** |
| **Ribosomal protein** | | | | |
| **+++** | **SPAC3F10.16c** | **lsg1^①^** | | **GTP binding protein, HSR1-related** |
| **+** | **SPAC959.08** | **rpl2102** | | **60S ribosomal protein L21** |
| **+** | **SPAC9G1.03c** | **rpl3001** | | **60S ribosomal protein L30** |
| **RNA binding protein** | | | | |
| **++** | **SPCC550.14** | **vgl1** | | **vigilin** |
| **++** | **SPCC74.09*** | **mug24*** | | **RNA-binding protein** |
| **+** | **SPCC757.09c** | **rnc1** | | **RNA-binding protein that suppresses calcineurin deletion Rnc1** |
| **Variety of other known functions** | | | | |
| **+++** | **SPAPB1E7.02c** | **mcl1** | | **DNA polymerase alpha accessory factor Mcl1** |
| **+++** | **SPBC106.07c** | **nat2** | | **N alpha-acetyltransferase Nat2** |
| **+++** | **SPBC365.14c** | **uge1** | | **UDP-glucose 4-epimerase** |
| **++** | **SPAC513.03** | **mfm2** | | **M-factor precursor Mfm2** |
| **++** | **SPAC17A5.14** | **exo2** | | **exonuclease II Exo2** |
| **++** | **SPBC14F5.13c** | **pho8^①^** | | **alkaline phosphatase** |
| **++** | **SPAC17G8.05** | **med20** | | **TATA-box related factor (TRF)** |
| **++** | **SPAC9G1.04** | **oxa101** | | **mitochondrial inner membrane translocase** |
| **++** | **SPCC736.07c** | **bud27/uri1** | | **cell polarity protein/unconventional prefoldin involved in translation initiation (predicted)** |
| **+** | **SPAC23H3.03c** | **npr2** | | **nitrogen permease regulator family** |
| **+** | **SPAC8F11.02c** | **dph3** | | **diphthamide biosynthesis protein Dph3** |
| **+** | **SPBC29A10.16c** | **cyb5^①^** | | **cytochrome b5** |
| **+** | **SPBPB10D8.04c** | **ssu1^①^** | | **membrane transporter** |
| **+** | **SPBC3H7.09** | **mug142** | | **palmitoyltransferase** |
| **+** | **SPBC16H5.06** | **rip1** | | **ubiquinol-cytochrome-c reductase complex subunit 5** |
| **+** | **SPAC26H5.05** | **mga2^①^** | | **IPT/TIG ankyrin repeat containing transcription regulator of fatty acid biosynthesis (predicted)** |
| **+** | **SPBC1709.09** | **rrf1** | | **mitochondrial translation termination factor** |
| **Unknown functions** | | | | |
| **+++** | **SPAC11G7.06c** | **mug132** | | ***S. pombe* specific UPF0300 family protein 3** |
| **++** | **SPCC613.03** | **ssp120^①^** | | **conserved fungal protein** |
| **+** | **SPAC3F10.05c** | **mug113** | | **DUF1766 family protein** |
| **+** | **SPCC594.02c** | **N/A** | | **conserved fungal protein** |
| **+** | **SPAC16E8.05c** | **mde1** | | **sequence orphan** |

**+++ indicates that the cells completely failed to grow on YPDA plus FK506 plates.**

**++ indicates that tiny colonies were observed to grow on YPDA plus FK506 plates.**

**+ indicates that colonies were observed on YPDA plus FK506 plates, however, the size of the** colonies were **significantly smaller than that of the wild-type cells.**

**indicates that the naming of genes is after the *S. cerevisiae* counterparts as the common name in *S. pombe* is not available.**

**N/A indicates that common gene name is not applicable.**

* **indicates poor growth on YPDA plates.**
